# Variability in CT lung-nodule quantification: Effects of dose reduction and reconstruction methods on density and texture based features

**DOI:** 10.1118/1.4954845

**Published:** 2016-07-29

**Authors:** P. Lo, S. Young, H. J. Kim, M. S. Brown, M. F. McNitt-Gray

**Affiliations:** Center for Computer Vision and Imaging Biomarkers, Department of Radiological Sciences, David Geffen School of Medicine, University of California, Los Angeles, California 90024

**Keywords:** computed tomography, texture, dose, reconstruction kernel, lung nodule, radiomics

## Abstract

**Purpose:**

To investigate the effects of dose level and reconstruction method on density and texture based features computed from CT lung nodules.

**Methods:**

This study had two major components. In the first component, a uniform water phantom was scanned at three dose levels and images were reconstructed using four conventional filtered backprojection (FBP) and four iterative reconstruction (IR) methods for a total of 24 different combinations of acquisition and reconstruction conditions. In the second component, raw projection (sinogram) data were obtained for 33 lung nodules from patients scanned as a part of their clinical practice, where low dose acquisitions were simulated by adding noise to sinograms acquired at clinical dose levels (a total of four dose levels) and reconstructed using one FBP kernel and two IR kernels for a total of 12 conditions. For the water phantom, spherical regions of interest (ROIs) were created at multiple locations within the water phantom on one reference image obtained at a reference condition. For the lung nodule cases, the ROI of each nodule was contoured semiautomatically (with manual editing) from images obtained at a reference condition. All ROIs were applied to their corresponding images reconstructed at different conditions. For 17 of the nodule cases, repeat contours were performed to assess repeatability. Histogram (eight features) and gray level co-occurrence matrix (GLCM) based texture features (34 features) were computed for all ROIs. For the lung nodule cases, the reference condition was selected to be 100% of clinical dose with FBP reconstruction using the B45f kernel; feature values calculated from other conditions were compared to this reference condition. A measure was introduced, which the authors refer to as *Q*, to assess the stability of features across different conditions, which is defined as the ratio of reproducibility (across conditions) to repeatability (across repeat contours) of each feature.

**Results:**

The water phantom results demonstrated substantial variability among feature values calculated across conditions, with the exception of histogram mean. Features calculated from lung nodules demonstrated similar results with histogram mean as the most robust feature (*Q* ≤ 1), having a mean and standard deviation *Q* of 0.37 and 0.22, respectively. Surprisingly, histogram standard deviation and variance features were also quite robust. Some GLCM features were also quite robust across conditions, namely, diff. variance, sum variance, sum average, variance, and mean. Except for histogram mean, all features have a *Q* of larger than one in at least one of the 3% dose level conditions.

**Conclusions:**

As expected, the histogram mean is the most robust feature in their study. The effects of acquisition and reconstruction conditions on GLCM features vary widely, though trending toward features involving summation of product between intensities and probabilities being more robust, barring a few exceptions. Overall, care should be taken into account for variation in density and texture features if a variety of dose and reconstruction conditions are used for the quantification of lung nodules in CT, otherwise changes in quantification results may be more reflective of changes due to acquisition and reconstruction conditions than in the nodule itself.

## 1. INTRODUCTION

Lung cancer remains the principal cause of cancer related deaths.^1^ Quantifying properties of lung nodules imaged on the CT for the purpose of diagnosis, staging, management, and determining treatment response has been a topic of interest for some time. Response evaluation criteria in solid tumors (RECIST),^2^ which assesses overall tumor burden via longest diameter of tumors and was originally intended for standardizing and simplifying tumor response criteria, has been accepted as a standardized measure of tumor response, especially in oncologic clinical trials. The NELSON study,^3^ which used a management protocol centered on the volume and volume doubling time of lung nodules, demonstrated how volumetric nodule assessment could be used routinely for managing patients with nodules detected through a lung cancer screening program.

There are also efforts that have described moving beyond the commonly used size based measures and into more sophisticated features that quantify the appearance and shape of lung nodules in CT via image processing and machine learning techniques. Aerts *et al.*^4^ showed the effectiveness of how tumor phenotypes can be quantified by applying a large number of quantitative image features, which they referred to as radiomics. El-Baz *et al.*^5^ proposed a spherical harmonics based shape index, which was computed on automatically segmented lung nodules that use an appearance and shape model, where they showed that their proposed measure was able to distinguish between malignant and benign lung nodules with high accuracy. Shen *et al.*^6^ proposed the use of multiscale convolutional neural networks to automatically extract discriminative features to differentiate between malignant and benign nodules. Han *et al.*^7^ compared the performance of 2D and 3D Haralick features in distinguishing between malignant and benign nodules via a support vector machine classifier. Thus, there are a number of other properties of lung nodules that may be extracted from CT image data which are being investigated as being helpful in diagnosis or patient management.

An ongoing concern in applying CT imaging techniques is the radiation dose to the patient. Thus, there have been steps toward using lower radiation dose techniques in CT imaging, both in screening as well as in diagnostic and followup CT examinations. In addition, the manufacturers have been developing many radiation dose reduction techniques such as automatic exposure control (AEC) methods and automatic tube current modulation (TCM),^8^ as well as advanced image reconstruction techniques such as iterative reconstructions that seek to reduce the image noise introduced when lower dose scans are used.^9^

It is well known that CT acquisition parameters impact appearance of structures and diseases in CT, but what is not clear is the effect of dose reduction methods on quantitative imaging measures derived from CT scans. Young *et al.*^10^ demonstrated that measured nodule volumes with semiautomated segmentation techniques were not sensitive to the effects of dose level and reconstruction kernel. Hunter *et al.*^11^ used 4D CT and 3D CT test–retest scans of non-small cell lung cancer cases to identify a set of nodule features that is robust across different CT machines. Mackin *et al.*^12^ investigated the effects of interscanner variability on radiomics features using a custom-designed phantom and found substantial variability in features derived from CT images when compared to patient cases with non-small cell lung cancer (NSCLC). Fave *et al.*^13^ found similar results when investigating radiomics features for patients imaged on cone beam CT. Except for Young *et al.*, the works above investigated inter or intrascanner variability of various features of lung nodules under similar CT acquisition and reconstruction conditions. There is still a need to investigate the effects of acquisition and reconstruction conditions on nodule density and texture based measures, both of which may be substantially impacted by reduced dose scans reconstructed with advanced image reconstruction techniques. This is similar to the approach described by Nyflot *et al.*^14^ who demonstrated the impact that stochastic effects have on quantitative texture features in PET-CT images.

The purpose of this work is to investigate the effects of dose level and reconstruction method on density and texture based features computed from CT lung nodules. A range of acquisition conditions is obtained both through repeat scanning of a phantom at different dose levels and through simulating reduced dose scans in patient image datasets. Compared to Mackin *et al.*,^12^ our analyses are limited to intrascanner variations due to different acquisitions and reconstruction conditions.

## 2. MATERIALS AND METHODS

This study consists of two major components. In the first component, an analysis of CT scans of a known uniform object, namely, a water phantom, was performed under a wide variety of acquisition (e.g., dose levels) and reconstruction (conventional filtered back projection or FBP and iterative reconstruction or IR) conditions. Because this object is completely homogeneous and uniform, any variability in the resultant images is therefore known to be due to the acquisition and reconstruction process. In the second component, a similar analysis of lung nodules from patient images was performed under a variety of acquisition and reconstruction conditions described previously in Young *et al.*^10^ To simulate a range of acquisition conditions, the raw projection data were obtained and noise was added using a validated method to simulate different dose levels. The original and simulated reduced-dose scans were reconstructed under several conditions. From the resulting image data, a range of density and texture values was extracted and analyzed to determine the effects of acquisition and reconstruction conditions on each feature value.

This section starts by describing the materials used in our experiments, which consists of CT scans of a water phantom and actual patients with lung nodules. Details on how reduced-dose scans were simulated and reconstructed at different kernels are presented. This is followed by a detailed description on the investigated density and texture features. Finally, the evaluation measures are presented.

### 2.A. Water phantom scans

Because patient nodules may not be homogenous in composition, we performed an initial set of experiments in homogeneous water phantom over a wide range of acquisition and reconstruction conditions to establish a basis for our investigation. The phantom used was the water section of the QA phantom of our CT scanner (Definition AS, Siemens Healthcare, Forchheim Germany). The water phantom was scanned on the Definition AS using variations of an adult abdomen/pelvis protocol under the following conditions: 120 kV, 0.5 s rotation time, pitch 1.0, collimation of 64 × 0.6, and fixed tube current scans of 225, 100, and 50 effective mAs, which corresponded to CTDIvol (32 cm phantom) of 17.1, 7.6, and 3.8 mGy, respectively. All images were then reconstructed at 1 mm thickness and 1 mm spacing using the eight different reconstruction kernels to represent a range of what is available on the scanner. These included conventional weighted FBP reconstructions of B10f (smoothest), B30f, B45f, and B70f (sharpest) kernels as well as IR (Safire) I26f strength 5 (smoothest), I44f strength 3, I50f strength 3, and I70f strength 1 (sharpest), designated as I26f∖5, I44f∖3, I50f∖3, and I70f∖1, respectively, which are somewhat analogous to the conventional reconstructions and used to illustrate a range of reconstruction options available on the scanner. These acquisition and reconstruction conditions are summarized in Table I.

**TABLE I.** Summary of acquisition and reconstruction conditions for the water phantom scans. Note that 17.1 mGy and B45 were the reference conditions here, indicated by “REF.” “X” indicates used reconstructions.

Dose level (mGy)	B10f	B30f	B45f	B60f	I26f∖5	I44f∖3	I50f∖3	I70f∖1
17.1	X	X	REF	X	X	X	X	X
7.6	X	X	X	X	X	X	X	X
3.8	X	X	X	X	X	X	X	X

### 2.B. Patient scans with nodules—Original and simulated reduced-dose images

A total of 33 cases with lung nodules from different patients were used in this study and were identical to the patient dataset used in Young *et al.*^10^ These nodules ranged in size from 7 to 46 mm longest in-plane diameter, with an average of 18 mm. Twenty-five nodules were >10 mm in diameter, and four of these were >30 mm in diameter. All scans were performed on a multidetector CT scanner (Definition Flash, Siemens Healthcare, Forchheim, Germany) using a routine, adult-chest protocol: 120 kV, 0.5 s rotation time, 250–285 quality reference mAs, pitch 1, with tube current modulation (TCM) (CareDose 4D, Siemens Healthcare, Forchheim, Germany). This is referred to as the “reference” protocol, which resulted in a CTDIvol of 20.9–23.8 mGy using the standard 32 cm CTDI body phantom.

As described previously, for each case, the original raw projection data were retrieved from the scanner so that simulated reduced dose scans would be created. This method is described in Young *et al.*^10^ and is based on the noise addition methods described by Zabic *et al.*,^15^ Massoumzadeh *et al.*,^16^ and Yu *et al.*^17^ Using this approach, we generated reduced-dose sinograms at 25%, 10%, and 3% of clinical dose. The TCM information is contained in the raw sinogram data. The TCM usually scales linearly with quality reference mAs setting and so dose reduction was modeled simply as a linear scaling of the TCM function by a constant factor. The reduced-dose TCM function was used to calculate the photon fluences for each projection in the helical scan trajectory, which was subsequently used as input to the noise-addition model.

After creating simulated reduced-dose sinograms, these sinograms were imported back to the scanner and reconstructed with three different reconstruction methods at each dose level: B45f, I44f strength 3 (I44f∖3), and I50f strength 3 (I50f∖3). These reconstruction methods would all be considered to be medium to medium sharp kernels and therefore represent a much narrower range of reconstruction conditions compared to those used on the water phantom scans. All images were reconstructed at 1 mm slice thickness and 1 mm interval. This is summarized in Table II.

**TABLE II.** Summary of acquisition and reconstruction conditions for the patient scans with lung nodules. Note that 100% dose (approx. 20.9 mGy) and B45f were the reference conditions here, indicated by REF. X indicates used reconstructions.

Dose level	B45ff	I44f∖3	B50f∖3
100% (20.9 mGy)	REF	X	X
25% (5.2 mGy)	X	X	X
10% (2.1 mGy)	X	X	X
3% (0.6 mGy)	X	X	X

### 2.C. Region of interest (ROI) and nodule contouring

For the water phantom, five nonoverlapping ROI of 10 mm diameter spheres were manually placed within the water region of the phantom from the reference condition (17.1 mGy, FBP with B45f kernel). For each nodule case, a single nodule was identified and semiautomatically contoured in 3D using the reference acquisition and reconstruction condition (100% clinical dose, FBP with B45f kernel) using an in-house software that used semiautomatic contouring, which was initiated via an Otsu thresholding^18^ to obtain an initial contour, where the user performs a click on a point within the nodule and a drag to a point outside the nodule. Additional regions were then added or erased manually via a paint interface until the desired contour was obtained. 17 randomly selected nodules were identified and contoured again using the same software. The lung nodule contours used in this study are a subset of contours used in Young *et al.*,^10^ which were contoured by three lab technologists trained in contouring lung nodules on CT scans.

### 2.D. Computation of density and texture based features

As mentioned earlier, we are interested in density and texture based features of lung nodules, which quantifies appearance of lung nodules based on CT densities (Hounsfield Units or HU). There is a very large family of features in the literature for quantifying the appearance of an object in an image. In addition, these features are also typically associated with a continuous parameter space, and can be used in combination to form new features. It is therefore not possible to exhaustively test all the image features in the literature nor is it the aim of this paper. Instead, we limit our scope to a subset of features belonging to two well known image feature families, which are histogram based features and gray level co-occurrence matrix (GLCM) based texture features. Histogram based features such as mean, standard deviation, and kurtosis of a given region of interest are some of the most commonly used density features not only in lung nodule quantification but also medical image quantification in general. GLCM based texture features, first introduced in Haralick *et al.*,^19^ are one of the most commonly used features for quantification of textures in image processing. Table III shows the list of features that were included in this study.

Computation of GLCM for a region of interest involves two parameters: the number of directions used (also referred to as offsets) and the number of quantization levels. In our experiments, we based the direction on the 26-connectivity that is typically used in 3D, resulting in a total of 13 offsets (=26/2 due to symmetry). This results in a total of 13 GLCMs computed for a given contour (one for each direction), resulting in a total of 13 values for a given GLCM feature. In practice, it is common to use the mean and range (maximum–minimum) across the different directions of a GLCM feature,^19^ which is what was done in this study. The number of quantization levels used is typically application specific and is usually chosen empirically. For this study, two quantization levels were used, which are 25^4^ and 32.

All computations were performed in 3D, which involves all voxels inside a given 3D contour. All feature values were computed from the contours obtained at the reference condition to prevent other sources of variations from influencing our analysis, such as intrareader variability and human perception of different reconstruction.

**TABLE III.** List of features included, where *I*, *P_I_*, and *P* are functions representing the image, normalize histogram, and normalized GLCM matrix of the image, respectively, and Ω is a set containing the coordinates of all voxels in the ROI.

Family	Feature	Formula
Histogram	Mean	$\mu =\left(\frac{1}{\left|\Omega \right|}\right)\underset{\vec{x}\in \Omega }{\sum }I\left(\vec{x}\right)$
	Median	
	Standard deviation	$\sigma =\left(\frac{1}{\left|\Omega \right|}\right)\sqrt{\underset{\vec{x}\in \Omega }{\sum }{\left(I\left(\vec{x}\right)-\mu \right)}^{2}}$
	Variance	*σ*^2^
	Skewness	$\left(\frac{1}{|\Omega |\sigma^{3}}\right)\underset{\vec{x}\in \Omega }{\sum }{\left(I\left(\vec{x}\right)-\mu \right)}^{3}$
	Kurtosis	$\left(\frac{1}{|\Omega |\sigma^{3}}\right)\underset{\vec{x}\in \Omega }{\sum }{\left(I\left(\vec{x}\right)-\mu \right)}^{4}$
	Entropy	$-\underset{i}{\sum }P_{I}\left(i\right)\log_{2}P_{I}\left(i\right)$
	Energy	$\underset{i}{\sum }P_{I}{\left(i\right)}^{2}$
GLCM	Mean	*μ_x_*
	Variance	(*σ_x_*)^2^
	Energy	$\sqrt{\alpha }$
	Entropy	$-\underset{i,j}{\sum }P\left(i,j\right)\ln P\left(i,j\right)$
	Contrast	$\underset{i,j}{\sum }{\left(i-j\right)}^{2}P\left(i,j\right)$
	Correlation	$\left(\frac{1}{\sigma_{x}\sigma_{y}}\right)\underset{i,j}{\sum }\left(ijP\left(i,j\right)-\mu_{x}\mu_{y}\right)$
	Dissimilarity	$\underset{i,j}{\sum }|i-j|P\left(i,j\right)$
	Homogeneity	$\underset{i,j}{\sum }\frac{P\left(i,j\right)}{1+{\left(i-j\right)}^{2}}$
	Information correlation A	$\frac{H_{xy}-H_{xy1}}{\max\left(H_{x},H_{y}\right)}$
	Information correlation B	$\sqrt{1-\exp^{-2\left(H_{xy2}-H_{xy}\right)}}$
	Maximum correlation coefficient	$\sqrt{\text{Second largest eigenvalue of}U}$
	Difference average	$\mu_{x-y}=\underset{i}{\sum }iP_{x-y}\left(i\right)$
	Difference variance	$\underset{i}{\sum }{\left(i-\mu_{x-y}\right)}^{2}P_{x-y}\left(i\right)$
	Difference entropy	$-\underset{i}{\sum }P_{x-y}\left(i\right)\log P_{x-y}\left(i\right)$
	Sum average	$\mu_{x+y}=\underset{i}{\sum }iP_{x+y}\left(i\right)$
	Sum variance	$\underset{i}{\sum }{\left(i-\mu_{x+y}\right)}^{2}P_{x+y}\left(i\right)$
	Sum entropy	$-\underset{i}{\sum }P_{x+y}\left(i\right)\log P_{x+y}\left(i\right)$
	Angular 2nd moment	$\alpha =\underset{i,j}{\sum }P{\left(i,j\right)}^{2}$
Definitions:		
	$\mu_{x}=\underset{i,j}{\sum }iP\left(i,j\right)$	
	$\mu_{y}=\underset{i,j}{\sum }jP\left(i,j\right)$	
	$\sigma_{x}=\sqrt{\underset{i,j}{\sum }{\left(i-\mu_{x}\right)}^{2}P\left(i,j\right)}$	
	$\sigma_{y}=\sqrt{\underset{i,j}{\sum }{\left(j-\mu_{y}\right)}^{2}P\left(i,j\right)}$	
	$P_{x}\left(i\right)=\underset{j}{\sum }P\left(i,j\right)$	
	$P_{y}\left(j\right)=\underset{i}{\sum }P\left(i,j\right)$	
	$P_{x+y}\left(k\right)=\underset{i,j}{\sum }P\left(i,j\right)\delta \left(k-\left(i+j\right)\right)$	
	$P_{x-y}\left(k\right)=\underset{i,j}{\sum }P\left(i,j\right)\delta \left(k-|i-j|\right)$	
	$\delta \left(i\right)=\{\begin{array}{ll} 1, & \text{if}\;k=0\\ 0, & \text{if}\;k\neq 0 \end{array}$	
	$H_{x}=-\underset{i}{\sum }P_{x}\left(i\right)\ln P_{x}\left(i\right)$	
	$H_{y}=-\underset{i}{\sum }P_{y}\left(i\right)\ln P_{y}\left(i\right)$	
	$H_{xy}=-\underset{i,j}{\sum }P\left(i,j\right)\ln P\left(i,j\right)$	
	$H_{xy1}=-\underset{i,j}{\sum }P\left(i,j\right)\ln\left(P_{x}\left(i\right)P_{y}\left(j\right)\right)$	
	$H_{xy2}=-\underset{i,j}{\sum }P_{x}\left(i\right)P_{y}\left(j\right)\ln\left(P_{x}\left(i\right)P_{y}\left(j\right)\right)$	
	$U\left(i,j\right)=\underset{k}{\sum }\frac{P\left(i,k\right)P\left(j,k\right)}{P_{x}\left(i\right)P_{y}\left(k\right)}$	

### 2.E. Analysis

#### 2.E.1. Water phantom

The role of the homogenous water phantom was to illustrate the change in feature values across conditions. The mean and standard deviation of each feature, computed across the five nonoverlapping sphere ROIs, were observed over the range of dose levels and reconstruction conditions. In order to identify conditions that are most similar to the reference condition, comparisons among conditions were performed based on individual feature values, with the nearest mean value to the reference condition being recorded and plotted in the form of a histogram.

#### 2.E.2. Patient scans with nodules

Similar to the previous analysis, the mean and standard deviation of each feature, computed across the different nodule cases, were used to illustrate the change in feature values across conditions.

The large variation of value range between different features makes it difficult to compare the effects of different conditions across the different features via simple measures such as mean squared difference. Therefore, a *Q* measure was used to quantify the robustness/stability of a feature *f* at a particular recon *r* with respect to the reference recon. The *Q* measure used is the ratio between the standard deviation of reproducibility (across acquisition and reconstruction conditions) and repeatability (across repeat contours of the reference condition), defined as

$$Q\left(f;r\right)=\frac{S\left(\left\{f_{r}\left(\Phi_{i}\right)-f_{0}\left(\Phi_{i}\right)|i=1,2,\ldots ,N\right\}\right)}{S\left(\left\{f_{0}\left(\Phi_{i}\right)-f_{0}\left(\Phi_{i}^{\prime }\right)|i=1,2,\ldots ,M\right\}\right)},$$ (1)

where *f_r_*(.) and *f*_0_(.) is the feature *f* computed for a given contour at reconstruction *r* and the reference recon, respectively, *S*(.) is a function that computes standard deviation, Φ_*i*_ and $\Phi_{i}^{\prime }$ are the contour of a case and its repeated counterpart, respectively, *N* is the total number of cases, and *M* is the total number of cases with a repeated contour. Intuitively, a feature *f* is said to be robust to reconstruction *r* if *Q*(*f*; *r*) ≤ 1. On the other hand, the larger *Q*(*f*; *r*) is from one, the more *f* is impacted by a change from the reference recon to reconstruction *r*.

## 3. RESULTS

### 3.A. Water phantom

**FIG. 1.**
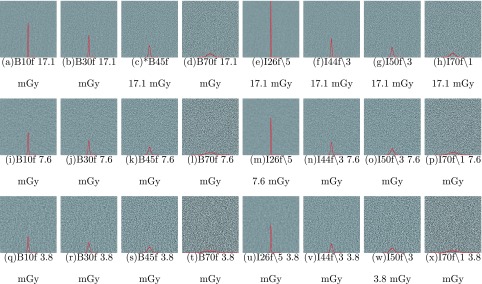
CT images of the water phantom obtained at eight kernels and three dose levels. The images show the same region of 125 × 125 mm cropped at the middle of the water phantom at window level of 0 and window width of 400. The overlaid red lines in the images are the histogram of the densities within cropped region. Both scales in the *X* and *Y* axis were kept constant across all images. The reference condition is indicated by *.

Figure 1 shows the cropped CT images of the water phantom at different kernels and dose levels, with the histogram of the densities (HUs) within the cropped region overlaid in the images. This figure demonstrates that both dose level and reconstruction condition have a substantial impact on local variation of HU values. It also shows, as expected, that the position of the peak of histograms did not vary much across reconstructions, indicating a stable mean value. The spread of the histogram however varies across conditions, having a smaller spread for the smoother kernels (lower reconstruction kernel numbers such as B10f) and a larger spread for the sharper kernels (higher numbers such as B70f). In addition, images using the same reconstruction condition, but different dose levels (i.e., images within the same column), illustrate the increased variation that can be observed as dose is reduced. For example, the differences can initially be appreciated based on visual comparisons between the smoothest image with the narrowest histogram peak, Fig. 1(e), to images with more variation and wider histograms, such as (d) that is of the same dose level, and (t) or (x) that are at reduced dose levels.

**FIG. 2.**
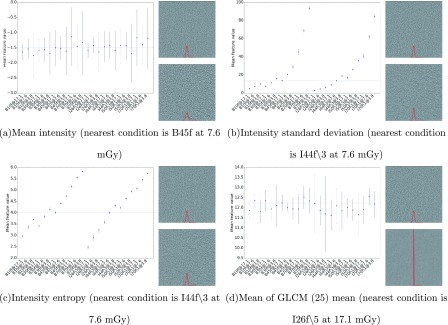
Plots of histogram features from a water phantom across different dose/reconstruction conditions, where the points and whiskers indicate the mean and the standard deviation of the feature value, respectively. The *y*-axes are the mean feature value at each condition and the *x*-axes are the various dose/reconstruction conditions. The images accompanying each plot are (from top to bottom) the image at the reference condition (B45f, 17.1 mGy) and the image from the condition having a mean feature value closest to the mean feature value at the reference condition. Note that the condition under which the closest match to the reference condition occurs changes with each feature used as the basis for the match.

To illustrate the effects of different dose level and reconstruction conditions on density and texture features, Fig. 2 shows the graph of a few selected features—the mean, standard deviation, entropy density, and mean of GLCM mean (at 25 quantization level) features across different conditions, where the point indicates the average feature values and the whiskers indicate the standard deviation across the ROIs. This figure illustrates that some features [e.g., mean, mean of GLCM mean (25)] are very stable across different conditions, while other features (e.g., standard deviation and entropy) vary substantially across conditions. Using the B45f kernel at 100% dose level as the reference condition, the image of the dose/reconstruction condition that has the mean feature value closest to the feature value under the reference condition was shown side by side with the reference image. This side-by-side visual and quantitative comparison is illustrated for each of the four features mentioned earlier in this paragraph.

**FIG. 3.**
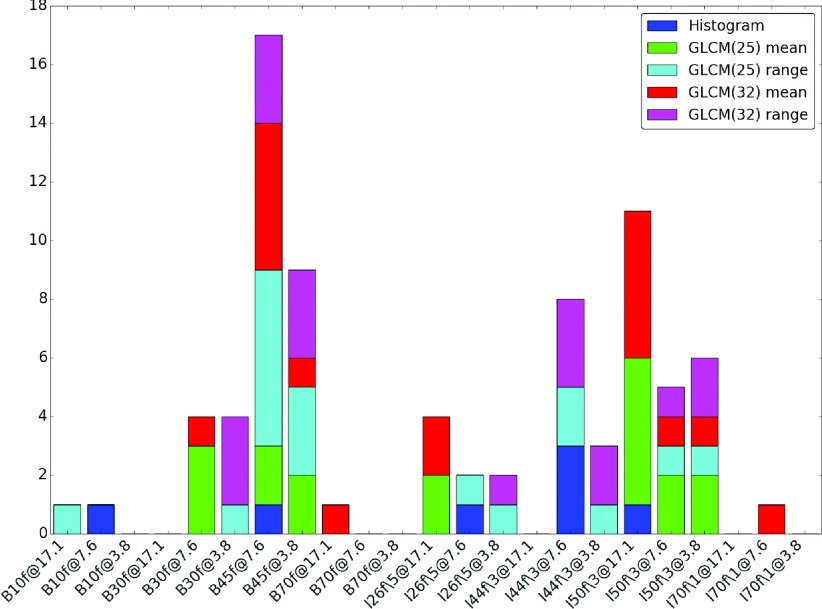
A histogram of how frequent a particular condition has the nearest mean feature value to that of the reference condition (B45f, 17.1 mGy) obtained using the water phantom images.

Figure 3 is a histogram of how frequent a particular condition has the nearest mean feature value to the reference condition. The majority of histogram features were observed to be closely matched to the I44f∖3 at 7.6 mGy condition, which more closely resembles (at least visually) the reference reconstruction. The majority of the GLCM features, however, were most closely matched to B45f at 7.6 mGy despite the difference in dose level with the reference condition.

### 3.B. Lung nodule cases

**FIG. 4.**
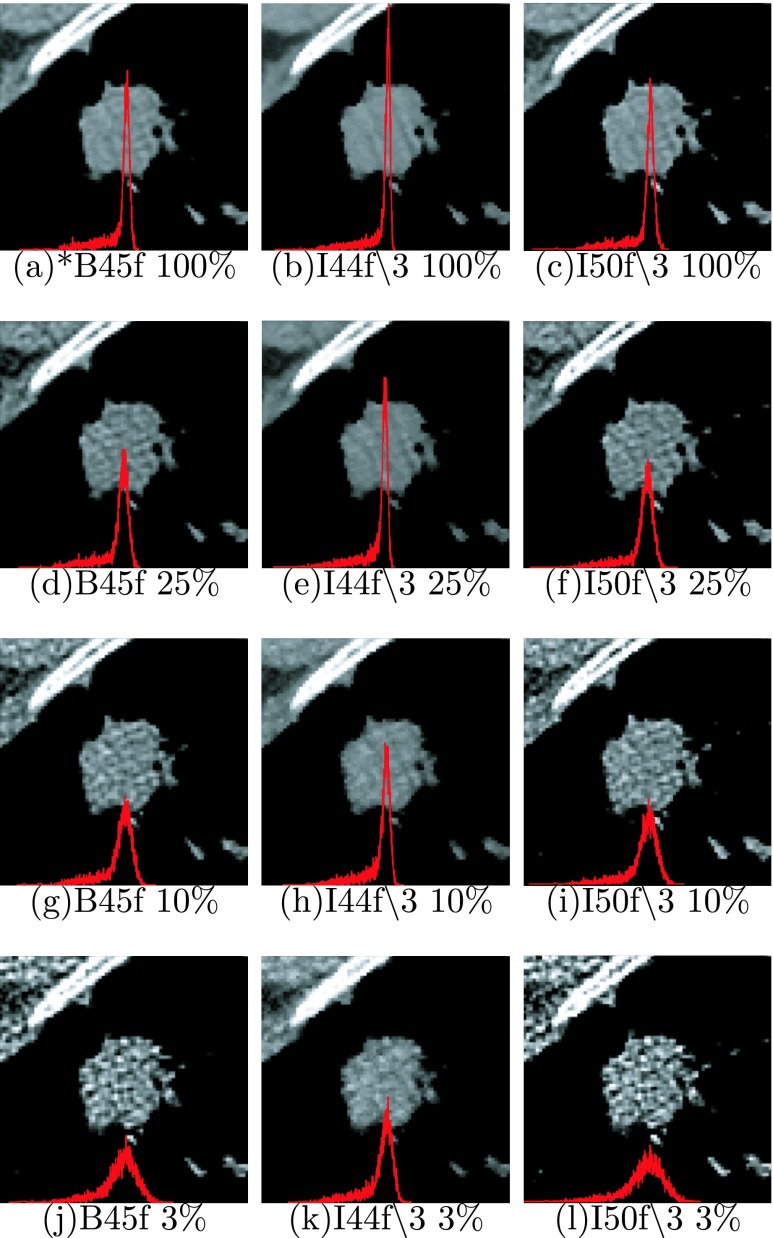
CT images of a nodule case obtained at three kernels and four dose levels (one original and three simulated), at a window level of 40 and window width of 400. The overlaid red lines in the images are the histogram of the densities within cropped region. Both scales in the *X* and *Y* axis were kept constant across all images. The reference condition is indicated by *.

A total of three simulated dose levels were generated from the raw sinogram data for each lung nodule case, which are 25%, 10%, and 3% of the original dose. For each raw sinogram datum (including both original and simulated), three reconstructions were obtained using FBP with B45f kernel and IR with I44f∖3 and I50f∖3, which represents a more limited range of reconstruction conditions compared to those used in the water phantom. This resulted in 12 reconstructions per lung nodule case. Figure 4 shows the CT images of the same nodule case with the different reconstructions. Overlaid on the images were the histogram of the densities of the nodule at the different reconstructions. Similar to the results of the water phantom, this figure demonstrates that both dose level and reconstruction condition have a substantial impact on local variation in HU values. Again, the differences between conditions can initially be appreciated based on visual comparisons between the smoothest image with the narrowest histogram peak, e.g., Fig. 4(b), to images with more variation and wider histograms, such as Fig. 4(j) or 4(l) which are both at simulated reduced-dose conditions.

**FIG. 5.**
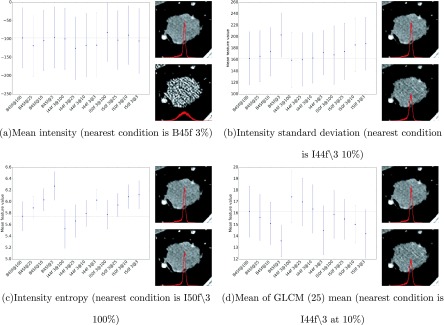
Plots of histogram features derived from nodules across different dose/reconstruction conditions, where the points and whiskers indicate the mean and the standard deviation of the feature value, respectively. The *y*-axes are the mean feature value at each condition and the *x*-axes are the various dose/reconstruction conditions. The images accompanying each plot are (from top to bottom) the image at the reference condition (B45f, 100%) and the image from the condition having a mean feature value closest to the mean feature value at the reference condition. Note that the condition under which the closest match to the reference condition occurs changes with each feature used as the basis for the match.

Figure 5 shows the graphs of the mean, standard deviation, entropy densities, and mean of GLCM mean (at 25 quantization level), similar to Fig. 2, of the contours at different conditions, where the point again indicates the average feature values and the whiskers indicate the standard deviation across the conditions. Using the B45f kernel at 100% dose level as reference, the image from the condition with a mean feature value that was closest to the mean feature value obtained at the reference condition was shown side by side with the reference image for several feature values. Because of the inhomogeneity of the nodule cases, the standard deviation of the features per condition is larger as compared to the ones from the water phantom (refer to Fig. 2). The influence of acquisition/reconstruction to histogram entropy and histogram standard deviation is also less prominent in the nodule cases as compared to those observed from the water phantom. There seems to be a consistent relationship between the mean of GLCM mean and the acquisition/reconstruction conditions for the nodule cases, which was not observed in the water phantom.

**FIG. 6.**
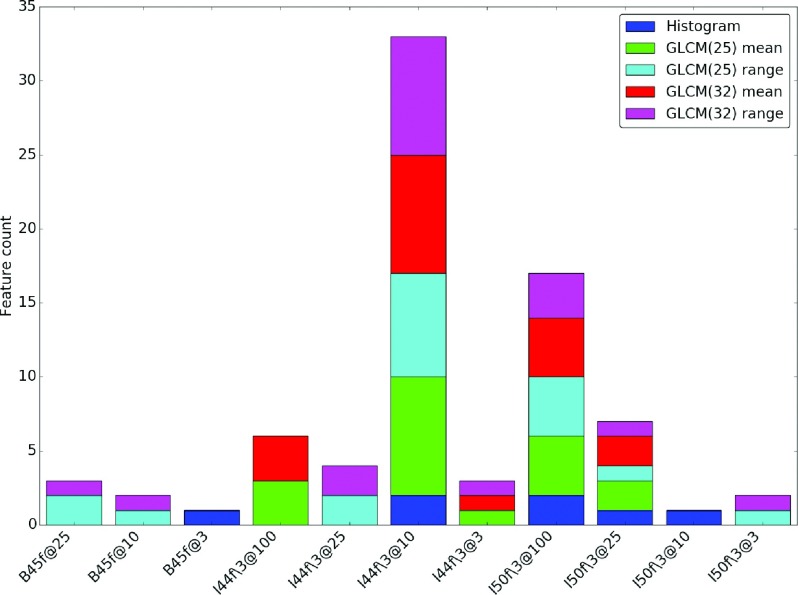
A histogram of how frequent a particular reconstruction has the nearest mean feature value to nearest to B45f at 100% from the nodule cases.

Figure 6 is a histogram of how frequent a particular condition has the nearest mean feature value to the reference condition. The results from the nodule cases show that the condition with the most frequent selection, which is I44f∖3 at 10%, is also the condition that appears visually to be the most similar to the reference condition, as observed in Fig. 4.

**FIG. 7.**
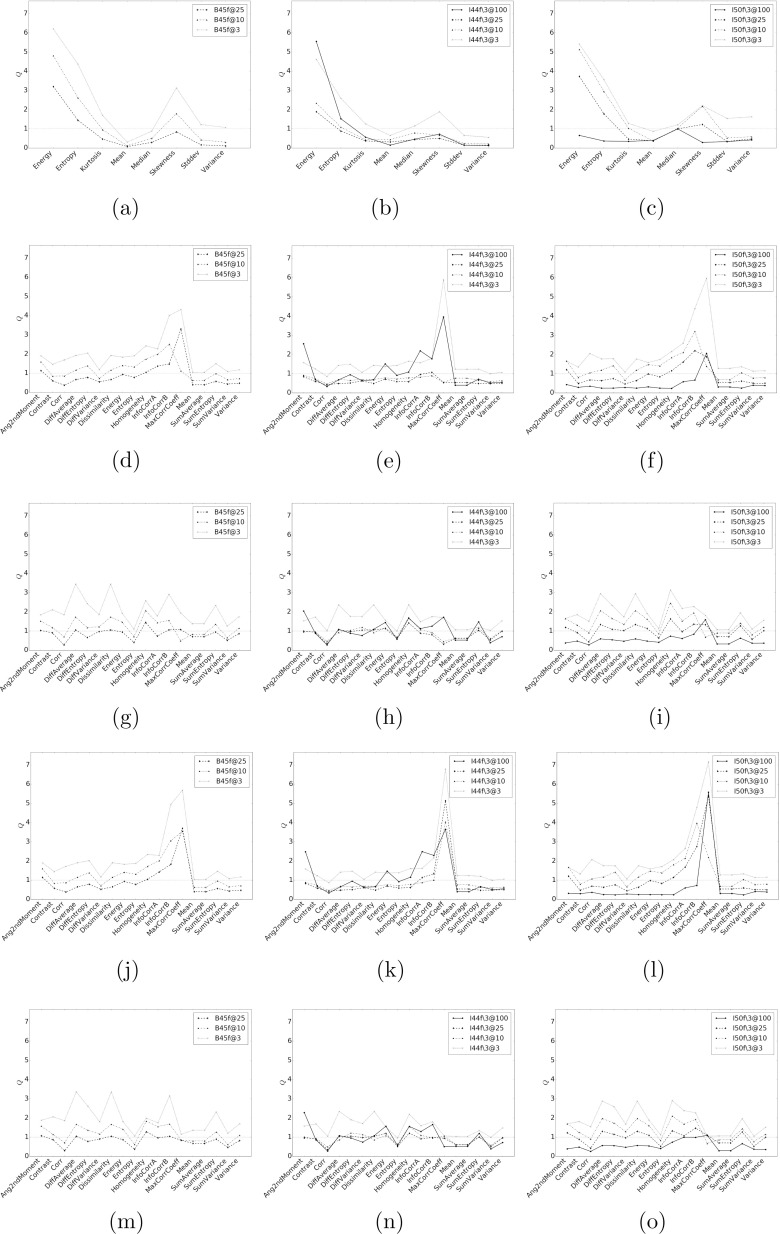
A plot of the stability measure *Q* (with respect to B45f at 100%) of the investigated histogram and texture based features across the dose/reconstruction conditions across the set of nodules. The *y*-axes are the *Q* values and the *x*-axes represent the features calculated at each dose/reconstruction condition. For each set of features, the figure in the first column represents the B45f reconstruction at 25%, 10%, and 3% dose levels; the second column represents the I44f∖3 reconstruction at 100%, 25%, 10%, and 3% dose levels; and the third column represents the I50f∖3 reconstruction at 100%, 25%, 10% and 3% dose levels. (a)–(c) are the histogram based features, (d)–(f) are the mean GLCM features computed at 25 levels, (g)–(i) are the GLCM feature range computed at 25 levels, (j)–(l) are the mean GLCM features computed at 32 levels, and (m)–(o) are the GLCM feature range computed at 32 levels for B45f, I44f∖3, and I50f∖3, respectively.

Figure 7 shows the evaluation measure *Q* for each of the histogram and texture measures with respect to the reference condition. As expected, the histogram mean feature is very robust to different conditions, even for the lung nodules. Similar to the observations in Figs. 5(b) and 5(c), the *Q* measure confirmed the sensitiveness of histogram entropy to acquisition/reconstruction conditions, and the relatively better robustness of histogram standard deviation.

Tables IV and V list the 30 lowest variation features and the 20 largest variation features across all conditions, respectively, ranked using the number of conditions with *Q* ≤ 1, and max *Q* in case of a tie. Histogram mean, variance, and standard deviation were the three features with the lowest variation. For GLCM features, both range and mean of sum variance, diff. variance, sum average and mean, and the range of entropy were among the 30 lowest variation features.

Table VI lists the rank of conditions according to the number of features with *Q* ≤ 1, and max *Q* in case of a tie, where I50f∖3@100% and I44f∖3@25% were the conditions with the lowest variation across all features. The three highest variation conditions were the conditions with 3% dose levels, with very few number of features with *Q* ≤ 1, especially for the I50f∖3 reconstruction in which only the histogram mean was robust.

## 4. DISCUSSION

In this study, we have investigated the behavior of histogram features and GLCM based texture features in both water phantom and nodule cases from actual patients across a variety of dose and reconstruction conditions. To avoid exposing patients to additional radiation, low dose scans were simulated from clinical dose CT raw data for the nodule cases. Due to the large amount of density and texture features available in the literature, and the near infinite possibilities of settings and combinations, exhaustive investigation is impossible. Instead, we limited the scope of our investigation to a subset of well known features that are used in lung nodule quantification related literature, which are histogram based features and GLCM based texture features.

As observed in Fig. 1, the appearance of a homogeneous object differs substantially across different acquisition and reconstruction conditions. From the histogram based features, it was observed that the mean HU value is quite robust to different conditions as expected, primarily because of CT numbers being consistently referenced and calibrated to water across all acquisition and reconstruction conditions. The main impact is on the spread or standard deviation, which is also observed from Figs. 2(a) and 2(b).

When comparing the features across different acquisition/reconstruction conditions with respect to a reference condition (100% clinical dose with FBP B45f reconstruction) as shown in Fig. 3, we observed that most histogram based features were nearest to I44f∖3 at 7.6 mGy. This condition is also one that resembles the reference condition visually, both appearance and histogram wise as shown in Fig. 1. However, the GLCM based texture features from the reference condition seemed to be nearest to B45f at 7.6 mGy and I50f∖3 at 17.1 mGy, which visually looks noisier than the reference condition. We suspect this is due to the quantization step in the computation of GLCM, as in I50f∖3 at 3.8 mGy and B45f at 7.6 mGy may appear to be more similar to the reference condition after quantization is applied.

As observed in Fig. 5, and similar to results observed from the water phantom, histogram mean of the nodule cases was quite robust across the different conditions. Surprisingly, unlike the water phantom, histogram standard deviation seems relatively robust. A possible explanation for this is that the variability introduced by the acquisition/reconstruction is masked by either the inhomogeneity in the nodule population or the inhomogeneity of densities within nodules as seen in the histogram shown in Fig. 4. While Fig. 3 showed different behavior between the histogram and GLCM features, the nodule cases showed general agreement of favored conditions (those conditions nearest in terms of mean feature value to the reference condition) between the histogram and GLCM features as observed in Fig. 6, where both types of features favor I44f∖3 at 10% and I50f∖3 at 100%.

As expected, HU mean is the feature with the lowest variation. This is followed by variance (rank 2) and standard deviation (rank 3). Other than GLCM mean of maximal correlation coefficient at 32 quantization level, all features have favored conditions with a *Q* measure of less than one, as shown in Tables IV and V.

In general, quantization level of 25 and 32 did not have much impact on the way the GLCM features behave. For the features belonging to the GLCM mean family, we did observe that features where their calculations involve summation of product between probability and bin index have lower variation in general, or of the form $\underset{i}{\sum }iP\left(i\right)$, such as sum variance, diff. variance, sum average, variance, and mean. With the exception of diff. variance, the same is true for GLCM range family. It is also surprising that while entropy of GLCM range was among the lowest variation features (rank 7 and 8 for quantization level of 25 and 32, respectively), entropy of GLCM mean was not.

Probably because of the limited range of different reconstructions in the nodule cases, effects due to different reconstructions appeared to be minor as compared to different dose levels. Conditions with dose level of 3% were the conditions that most features (with the exception of histogram mean) were sensitive to, with little or no features having *Q* ≤ 1. It is interesting that I44f∖3 at 100% dose level has less features with *Q* ≤ 1 as compared to 25% and 10% dose levels, indicating that additional noise from the lower dose levels actually helps in countering the smoothing effect of the reconstruction method.

Table VI shows the rank of the different conditions according to the number of robust features (*Q* ≤ 1). While the majority of features are robust (with respect to the reference condition) for high ranking conditions (e.g., I50f∖3 at 100%), there are still sensitive features with *Q* > 3. This implies that if these sensitive features were used to quantify nodules, care must be taken to ensure that the acquisition conditions are the same (e.g., the reference condition in our case), for instance at different time points, or else changes in the quantification results may reflect the difference in acquisition conditions rather than the underlying physiology.

One limitation of this study was that the patient nodule scans were not analyzed under the same conditions as the water phantom, thereby limiting some of the direct comparisons between the water phantom and nodule results. This was in large part due to an equipment change such that the scanner on which the original patient data were acquired (Definition Flash) was removed and no longer available for reconstructions (the raw projection data from the Flash could not be reconstructed on any other nonFlash scanner). This limited the range of conditions under which the patient nodule data could be analyzed. The water phantom was scanned on a similar but not identical multidetector CT (Definition AS) and reconstructions were performed under a wider range of conditions to more completely illustrate the effects of dose and reconstruction on feature calculations for a homogeneous object.

**TABLE IV.** 30 lowest variation features extracted from the lung nodules according to the number of conditions with *Q* ≤ 1, and max *Q* in case of a tie.

Rank	Family	Feature	Mean *Q*	Standard deviation *Q*	Min *Q*	Max *Q*	# *Q* ≤ 1(12)	Max condition
1	Histogram	Mean	0.37	0.22	0.08	0.87	11	I50f∖3@3
2	Histogram	Variance	0.51	0.44	0.11	1.62	9	I50f∖3@3
3	Histogram	Stddev	0.52	0.44	0.15	1.54	9	I50f∖3@3
4	GLCM(32) range	Sum variance	0.68	0.27	0.37	1.2	9	B45f@3
5	GLCM(32) mean	Sum variance	0.69	0.25	0.4	1.13	9	I50f∖3@3
6	GLCM(25) mean	Sum variance	0.69	0.26	0.4	1.13	9	I50f∖3@3
7	GLCM(25) range	Entropy	0.74	0.24	0.4	1.12	9	I50f∖3@3
8	GLCM(32) range	Entropy	0.69	0.25	0.37	1.12	9	I50f∖3@3
9	GLCM(32) mean	Correlation	0.84	0.55	0.33	2.07	8	I50f∖3@3
10	GLCM(25) mean	Correlation	0.83	0.55	0.33	2.05	8	I50f∖3@3
11	GLCM(32) range	Correlation	0.74	0.52	0.26	1.86	8	B45f@3
12	GLCM(25) range	Correlation	0.73	0.51	0.27	1.85	8	B45f@3
13	GLCM(25) mean	Contrast	0.83	0.35	0.29	1.46	8	B45f@3
14	GLCM(32) mean	Contrast	0.82	0.35	0.3	1.45	8	B45f@3
15	GLCM(25) range	Mean	0.79	0.28	0.32	1.38	8	B45f@3
16	GLCM(25) range	Sum average	0.79	0.28	0.32	1.38	8	B45f@3
17	GLCM(32) range	Sum average	0.78	0.28	0.29	1.36	8	B45f@3
18	GLCM(32) range	Mean	0.78	0.28	0.29	1.36	8	B45f@3
19	GLCM(25) mean	Sum average	0.71	0.32	0.31	1.27	8	I50f∖3@3
20	GLCM(25) mean	Mean	0.71	0.32	0.31	1.27	8	I50f∖3@3
21	GLCM(32) mean	Mean	0.71	0.32	0.31	1.27	8	I50f∖3@3
22	GLCM(32) mean	Sum average	0.71	0.32	0.31	1.27	8	I50f∖3@3
23	GLCM(25) range	Sum variance	0.71	0.28	0.36	1.26	8	B45f@3
24	Histogram	Median	0.79	0.3	0.29	1.21	8	I50f∖3@3
25	GLCM(25) mean	Diff variance	0.73	0.27	0.28	1.19	8	B45f@3
26	GLCM(25) mean	Variance	0.72	0.27	0.38	1.18	8	B45f@3
27	GLCM(32) mean	Variance	0.72	0.27	0.38	1.17	8	B45f@3
28	GLCM(32) mean	Diff variance	0.71	0.26	0.28	1.16	8	B45f@3
29	Histogram	Kurtosis	0.81	0.44	0.35	1.7	7	B45f@3
30	GLCM(25) mean	Sum entropy	0.86	0.38	0.25	1.5	7	B45f@3

**TABLE V.** 20 highest variation features extracted from the lung nodules according to the number of conditions with *Q* ≤ 1, and max *Q* in case of a tie.

Rank	Family	Feature	Mean *Q*	Standard deviation *Q*	Min *Q*	Max *Q*	# *Q* ≤ 1(12)	Max condition
61	GLCM(32) range	Angular 2nd moment	1.39	0.5	0.39	2.28	3	I44f∖3@100
62	GLCM(25) range	Angular 2nd moment	1.34	0.46	0.37	2.04	3	I44f∖3@100
63	GLCM(25) range	Diff variance	1.21	0.41	0.47	1.86	3	B45f@3
64	GLCM(25) mean	Maximal correlation coefficient	2.81	1.91	0.54	5.97	2	I50f∖3@3
65	GLCM(25) mean	Information correlation B	2.18	1.18	0.67	4.39	2	I50f∖3@3
66	Histogram	Entropy	2.11	1.16	0.36	4.37	2	B45f@3
67	GLCM(25) range	Diff average	1.69	0.87	0.59	3.44	2	B45f@3
68	GLCM(25) range	Dissimilarity	1.69	0.87	0.59	3.44	2	B45f@3
69	GLCM(32) range	Dissimilarity	1.65	0.85	0.57	3.36	2	B45f@3
70	GLCM(32) range	Diff average	1.65	0.85	0.57	3.36	2	B45f@3
71	GLCM(32) mean	Information correlation A	1.72	0.64	0.6	2.65	2	I50f∖3@3
72	GLCM(25) range	Sum entropy	1.34	0.45	0.62	2.33	2	B45f@3
73	GLCM(32) range	Sum entropy	1.31	0.45	0.66	2.31	2	B45f@3
74	GLCM(25) range	Energy	1.34	0.41	0.47	1.92	2	B45f@3
75	GLCM(32) range	Energy	1.33	0.39	0.54	1.88	2	I50f∖3@3
76	Histogram	Energy	3.96	1.67	0.66	6.2	1	B45f@3
77	GLCM(32) mean	Information correlation B	2.62	1.38	0.72	4.94	1	B45f@3
78	GLCM(25) range	Homogeneity	1.93	0.64	0.73	3.13	1	I50f∖3@3
79	GLCM(32) range	Homogeneity	1.7	0.56	0.74	2.9	1	I50f∖3@3
80	GLCM(32) mean	Maximal correlation coefficient	4.8	1.44	2.19	7.16	0	I50f∖3@3

**TABLE VI.** Ranking of conditions according to the number of features with *Q* ≤ 1, and max *Q* in case of a tie. The number in square brackets indicates the total number of features in the indicated feature family.

							# *Q* ≤ 1			
Rank	Reconstruction	Dose level	Mean *Q*	Standard deviation *Q*	Min *Q*	Max *Q*	Total	Intensity [8]	GLCM(25) [36]	GLCM(32) [36]
1	I50f∖3	100	0.54	0.64	0.23	5.58	76	8	34	34
2	I44f∖3	25	0.78	0.58	0.15	5.13	63	7	29	27
3	I44f∖3	10	0.86	0.47	0.22	4.03	62	6	29	27
4	B45f	25	0.87	0.6	0.08	3.7	57	6	25	26
5	I44f∖3	100	1.08	0.89	0.13	5.55	49	6	21	22
6	I50f∖3	25	1.03	0.73	0.34	5.38	46	5	20	21
7	B45f	10	1.26	0.71	0.12	4.81	33	5	14	14
8	I50f∖3	10	1.4	0.76	0.37	5.13	27	3	12	12
9	I44f∖3	3	1.59	0.94	0.56	6.79	10	3	3	4
10	B45f	3	2.04	1.05	0.31	6.2	2	2	0	0
11	I50f∖3	3	1.98	1.11	0.87	7.16	1	1	0	0

## 5. CONCLUSION

We have illustrated the effects of dose and reconstruction (both FBP and IR) methods have on density and texture based features computed from CT scans of a uniform water phantom and nodule cases. Even though acquired under similar acquisition conditions, the effects of the features on a uniform water phantom differ as compared to those from the nodule cases. Though only at a limited number of dose levels and reconstructions, we showed that using lower dose level (more noisy) on smoother reconstruction, or vice versa, may have a balancing effect and results in images and feature values similar to images acquired from other conditions.

It was shown that different features were impacted differently by different dose level and reconstruction, and some features are more robust to different conditions than others. Additionally, we observed that most features computed based on summation of the product of probability and intensity values (either in HU or values after quantization) have a tendency to be more robust to different acquisition conditions, barring a few exceptions.

It should be noted that our focus in this study is on reproducibility of features across different acquisition conditions, which may or may not relate to physiology of lung nodules, for instance prognosis of nodule malignancies.

In conclusion, when using densities or texture features for quantification of nodule physiology, care should be taken to take account of the susceptibility of the features used to the physics-based variation, either through careful control of acquisition conditions or the usage of more robust features. If such account is not taken into consideration, the quantification results may reflect more to the acquisition conditions rather than the actual nodule physiology, resulting in inaccurate prognosis or diagnosis.
